# Two Independent Prospectively Planned Blinded Weibull Statistical Analyses of Flexural Strength Data of Zirconia Materials

**DOI:** 10.3390/ma9070512

**Published:** 2016-06-24

**Authors:** Malgorzata Roos, Christine Schatz, Bogna Stawarczyk

**Affiliations:** 1 Department of Biostatistics, Epidemiology Biostatistics and Prevention Institute, University of Zurich, Hirschengraben 84, 8001 Zurich, Switzerland; 2 Department of Prosthodontics, Dental School, Ludwig-Maximilians-University Munich, Goethestrasse 70, 80336 Munich, Germany; c.sa.schatz@googlemail.com (C.S.); bogna.stawarczyk@med.uni-muenchen.de (B.S.)

**Keywords:** flexural strength data, Weibull analysis, blinded statistical analysis, permutation tests

## Abstract

Zirconia as a restoration dental material are gaining attention because of their high mechanical properties and good biocompatibility. Therefore, investigation of the flexural strength of zirconia is of great interest. For this purpose, Weibull statistics for description of the material reliability are frequently used. The aim of this work was to present a blinded data set to two independent statisticians for two parallel statistical analyses in order to find an optimal statistical approach for analysis of in-vitro measured flexural strength data of zirconia materials. A prospectively planned independent blinded statistical analysis implementing three quality control actions “blinded data set”, “independent statistical analyses” and “parallel manuscript writing” was designed. Statistical analysis paths taken by both biostatisticians differed. They arrived at complementary results. The major difference was caused by two alternative distributional assumptions (Weibull/Normal) and alternative fitting methods (LS/ML). The parallel statistical analysis and manuscript writing approach on a blinded data set greatly supported our choice of statistical methods for analysis of flexural strength results of zirconia materials.

## 1. Introduction

Monolithic zirconia was introduced to avoid the risk of dental restoration failure due to veneer-chipping fractures. To improve the esthetic appearance of zirconia restorations, attempts were made to improve translucency. One way was to increase the sintering temperature leading to microstructural changes in the material. As literature shows this method has a significant drawback. Grain growth and a non-homogenous structure influences the flexural-strength negatively [1]. Hence the Al_2_O_3_ percentage was reduced from 0.25 to 0.05 wt % and the smaller Al_2_O_3_ grains were positioned on the boundaries of the zirconia grains, which also results in a higher translucency of the material. At this moment, insufficient information is available about the flexural strength of second generation zirconia. The reliability of these flexural strength findings gives further insight to the performance and is an indicator of a zirconia material’s quality. For this purpose, Weibull statistics are used. However, Weibull statistics can lead to minimal deviations depending on the estimation method [2]. The aim of this work was to present the blinded data to two statisticians in order to see how and if the further statistical investigation leads to a consensus view of the quality of the zirconia materials. The blinded data set provided for the parallel statistical analysis contained 240 measurements from a previous study [3] on biaxial flexural strength for three monolithic zirconia materials (ZM) Ceramill Zolid, Zenostar ZrTranslucent and DD Bio zx2 for which two different specimen preparation (SP) methods: either dry polishing before sintering or wet polishing after sintering were applied.

### 1.1. Motivation for a Weibull Analysis

In dentistry, brittle materials are well characterized using Weibull statistics [4,5]. Ceramics, especially the high-performance ceramics such as zirconia or alumina are standardly tested for the reliability and homogeneity of the structure of the material by Weibull modulus (m).

For statistical analysis of flexural strength data [2,6,7,8] both the two-parameter Weibull (s, m) and the two-parameter Normal (mean, sd^2^) distributional assumptions are frequently used. Weibull modulus m describes the reliability of the measurements with higher values corresponding to better reliability of the material. Normal mean corresponds to the characteristic strength (scale, s) of the Weibull distribution. Weibull modulus (m) is approximately inverse proportional to the Normal standard deviation (sd) (m = 1/sd). A reliable material with a high Weibull modulus (m) has low values of standard deviation (sd) for Normal distributional assumption as sd = 1/m. When analyzing data both differences of s (mean) and m (sd) estimates between the different groups for Weibull (Normal) distributional assumption are of interest.

Application of Normal and Weibull distributional assumptions implies a different perception of the underlying truth. First, the Normal distribution emerges when each observation is a sum of a possibly large number of independent random fluctuations [9]. In contrast, the stochastic process governing the Weibull distribution follows the “weakest link” concept [10,11,12]. Internal independent flaws propagate under external stress and eventually a specimen breaks at the weakest place. This way a single flaw dictates the strength of the whole system. Second, the support of the Normal distribution comprises the whole real line. Consequently, it is not perfectly suitable for modelling positive flexural strength observations. In this respect the Weibull distribution is more appropriate as it is defined on the positive real line. The third argument in favour of the Weibull distribution is its shape flexibility. Weibull distribution is capable of modelling symmetric as well as negatively and positively skewed data. In contrast, the Normal distribution is only acceptable for symmetric data.

Although the probabilistic basis for application of the Weibull distribution for brittle materials was quite strong its utilization in practice was hampered by a fairly cumbersome parameter estimation [4,10]. Recently, much progress has been made with respect to the availability of the Weibull distribution. There are flexible general-purpose statistical programs for estimation of the two-parameter Weibull distribution applying either the maximum likelihood (ML) or the least squares (LS) methodologies or both [6]. Many of them provide 95% confidence intervals for the Weibull parameters, convenient probability plots and conduct tests for parameter differences between factor levels. There is also a free available open source Excel-calculator facilitating an automatic LS estimation of Weibull parameters together with the corresponding 95% CI (Appendix C in [13]). Aside of this eminent progress there is still an uncertainty caused by a data analyst [14].

### 1.2. Motivation for a Prospectively Planned Independent/Parallel Blinded Statistical Analysis

A typical study passes through three work phases consisting of data generation, statistical analysis and manuscript writing (Table 1, study phases a–c). Each phase can be afflicted by several sources of uncertainty [14], which can be intentional or unconscious. Various problems in design, conduct, analysis and reporting of research may lead to biases toward findings that can distort the perception of the research progress [15].

Several authors [14,16,17] warn that an objectivity of statistical data analysis (Table 1, study phase b) is a clear misperception. The pretended objectivity of data analysts can be affected adversely [17] by many factors. For example, a statistician’s bias is caused by differences in available statistical tools, techniques, programs and personal experience. In the course of statistical data analysis numerous semi-subjective decisions (Table 1, study phase b) have to be made that can have major effects on the results of the study [14,16]. Such semi-subjective decisions include assumptions to be made and models to be applicable [14], finally leading to the choice of a particular statistical method. Subjectivity is also involved in performing the analysis and interpreting the results. Perception of the data and the choice of the analysis can considerably vary across statisticians. A bias in direction of preferred methods, prior theoretical expectations or some other preconceptions is possible and very likely [14].

Usually, the size of the statistician’s bias cannot be estimated. Therefore, it is beneficial to use methodology that suppresses, prevents or indicates it clearly. One possible resort is the use of the blinded design [18,19,20]. Blinding is a research strategy that involves the deliberate withholding of information from people who play a role in a study [16,21,22]. In principle, blinding of subjects, investigators, outcome assessors, data managers, biostatisticians and manuscript writers or any combination of them is possible [16,21,22,23]. The idea of blinding is a well-known scientific method frequently used in many fields of research and notably in the context of clinical trials [18,19,23,24]. The wide ranging applicability of the blinding technique guarantees its validity not only in in-vivo but also in in-vitro studies. For example, it is extensively used in modern statistical analysis of particle physics experiments [25], where measurements and conducted experiment are completely unknown to the involved analysts.

Whereas single- and double-blinded study designs concentrate mainly on reduction of uncertainties at the data generation step (Table 1, study phase a), by blinding the subjects only and both subjects and investigators, respectively, the statistical data analysis phase (Table 1, study phase b) has been perceived as an objective one, especially if the statistician is not involved in the experimental process [18]. This belief, however, clearly disagrees with observation that both statistical analysis and writing of the manuscript are potential sources of bias that persist even in double-blinded trials [17].

To counteract these problems a triple-blinded design has been suggested [23,24]. The triple-blinded design—a double-blind trial that also maintains a blind data analysis—aims for reduction of a potential bias introduced by the statistical data analysis [23,24] by keeping the involved analysts in dark about the meaning of the treatment groups specification during data analysis. In such a case the analyst is blinded to the meaning of the data. Ideally, data entry is done independently of the analyst and codes for treatment group assignments are allocated randomly [16]. The “blinded data set” approach aims for objective and straight decisions during the conduct of statistical analysis.

Unfortunately, an analysis of a blinded data set can be still insufficient for the bias reduction induced by the statistician. Therefore, Polit [16] and Miller & Stewart [23] suggest different strategies for broadening of the blinding technique within the statistical analysis phase (Table 1, study phase b). They claim that a blinded data analysis is most efficiently achieved by an “independent statistical analysis” when two independent data analysts are involved and both are blinded to the treatment group status. In a scenario when two statisticians are analyzing the same data set, free communication between them could promote convergence towards a consensus and important clues might be missed. The respective final analyses might be distorted and the results biased. Hence, in order to fully profit from a parallel data analysis, statisticians should analyze the data independently and provide their stand-alone description of the outcomes. Keeping this in mind Gøtzsche [17] suggests application of independent investigation not only within statistical data analysis phase (Table 1, study phase b) but also during the process of manuscript writing (Table 1, study phase c). In particular, “Results”, “Summary”, “Conclusion” and “Rationale for the choice of the statistical methods” sections should be drafted in two independent versions [17] leading to a “parallel manuscript writing” approach. Moreover, any additional analyses, performed after code breaking, should be identifiable as such. Hence, any independent blinded statistical analysis which joins both “independent statistical analyses” and “parallel manuscript writing” techniques and applies them to a “blinded data set” aims for diminishing not only the outcome reporting bias but also the risk that the conclusion and treatment recommendation are influenced by irrelevant factors [17].

Blinding of the data analyst has been identified as an easy way to minimize bias and to enhance credibility of the results [16]. The blinded data strategy initiated by Gøtzsche [17] has been applied in an increasing amount of studies. Depending on the field of research data analysts are blinded in as much as 2.5%–15% of research projects [16]. In each case, a blinded design seeks to achieve a higher standard of scientific rigor than a conventional non-blinded one.

Although over the years blinded analysis strategy has been steadily gaining in importance in numerous fields of research, its application in the context of dental materials research is still lacking. Therefore, we prospectively designed an independent blinded statistical analysis which could be conveniently applied for dental material projects. The aim of this study was to investigate by means of two independent prospectively planned blinded analyses if there is an agreement in Weibull statistics of flexural strength data of different zirconia ceramics analyzed independently by two statisticians using differing statistical approaches. We applied a random allocation of coding to tested groups in the data set and kept the analysts in dark about their meaning. What is more, any communication between statisticians during this time period was forbidden. Two independent drafts describing findings and statistical methods were written before code breaking.

The first hypothesis for the dental material research states that monolithic zirconia specimen preparation (SP) has no impact on the flexural strength. The second hypothesis states that all three tested zirconia materials (ZM) show similar flexural strength results. Additionally, two hypotheses with respect to two independent prospectively planned blinded Weibull statistical analyses were formulated. First, there is an agreement in statistical analysis paths chosen by both biostatisticians for Weibull analysis. Second, the three quality control actions: “blinded data set”, “independent statistical analyses” and “parallel manuscript writing” conducted by two independent biostatisticians (implemented within our prospectively planned parallel blinded statistical analysis) have no influence on findings for flexural strength data.

## 2. Material and Methods

### 2.1. Experimental Data Description

Data for blinded analysis were a subset of a larger data collection in [3]. Data provided to the statisticians by the study supervisor A consisted of 240 biaxial flexural strength measurements divided randomly into six groups G1–G6. Each group was defined by a factor zirconia material (ZM with three levels C = Ceramill Zolid, Z = Zenostar ZrTranslucent and D = DD Bio zx2) and specimen preparation (SP with two levels before = dry polishing before sintering, after = wet polishing after sintering) and contained 40 specimens (Table 2). The specimen preparation process consisted of the following steps. The specimens were cut out of zirconia cylinders with a low speed diamond saw (Well, Diamantdrahtsägen, Mannheim, Germany). Manual dry polishing was conducted with SiC discs (Struers, Ballerup, Denmark) and machine wet polishing after sintering was executed with a water-cooled polishing machine (Struers Abramin, Struers, Ballerup, Denmark). For the sintering process a universal sintering oven (Nabertherm, Lilienthal/Bremen, Germany) was used. Only one operator handled the specimens. The final dimension DIN EN ISO 6872:2008 [26] of all disc shaped specimens was 16 mm × 1.2 mm (±0.05 mm). For biaxial flexural strength measurement, the specimen were put on to a sample holder, which consisted of three tempered steel balls (diameter 3.2 mm) forming an equilateral triangle (edge length 10 mm and ball support circle 120°). The plunger (diameter 1.4 mm) of the Universal Testing Machine (Zwick, Ulm, Germany) loaded the specimens with a crosshead speed of 1 mm/min until failure.

### 2.2. A prospectively Planned Independent Blinded Statistical Analysis

**Figure 1:**
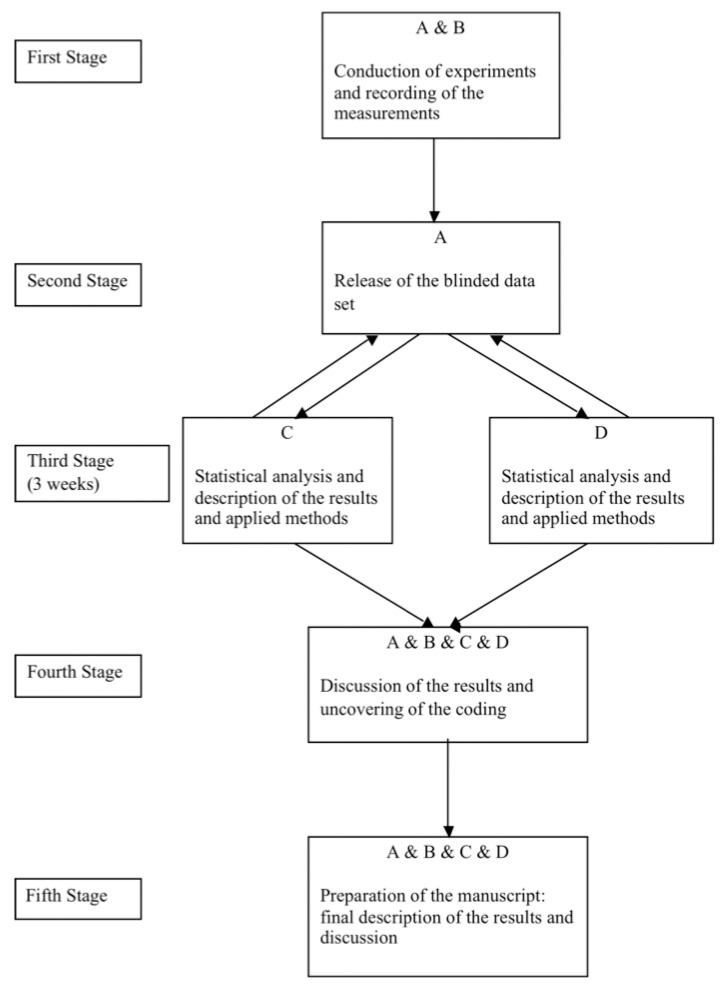
Plan of the independent blinded statistical analysis.

The independent blinded statistical analysis technique was devised prior to the data analysis. Figure 1 depicts the details of our plan, which consisted of five stages. For sake of clarity of presentation, the contributors in the sequel are identified by capital letters A, B, C and D. In the planning stage of our study the contributor A was designated to be the supervisor of the project and had an unlimited insight in all phases of the study.

In the first stage contributors A and B were responsible for conduction and execution of experiments and recording of the measurements in an Excel file. In the second stage A randomly assigned coding numbers to the tested groups, kept the key identifying the meaning of the factors in the data set and was not allowed to reveal it to both statisticians’ C and D until the statistical analysis of the measurements was accomplished. In addition, contributor A checked graphically the plausibility of recorded measurements and correctness of factors coding.

Beginning of the third stage was marked by the release of the blinded data set to statisticians by contributor A. Both statisticians’ C and D were not allowed to communicate and interact with each other during this stage. In case of any questions they might, however, address the supervisor A directly. They were given 3 weeks to accomplish statistical analysis of the data and to describe their findings and statistical methods applied with all tables, graphs and references necessary to back up their conclusions. During this time, they wrote two independent drafts of “Results”, “Summary”, “Conclusion” and “Rationale for the choice of the statistical methods” sections. Apart of that the time amount needed for accomplishing every step of the statistical analysis and draft writing was recorded. The statisticians were blinded to the meaning of data coding and their knowledge about the goal of statistical analysis was limited to a short note provided by A accompanying the data set.

Only after all data had been analyzed and two independent drafts had been written the fourth stage consisting of a meeting attended by all involved contributors (A, B, C, D) was scheduled. During this meeting not only written exposition of the results provided independently by C and D and applied statistical methods were compared with each other but also possible reasons for the discrepancies in findings were discussed. The amount of time invested in analysis and draft writing was compared. Eventually, A resolved the key for the meaning of the factor levels and the primary outcome in the data making the results interpretable. In case of major differences in results the following procedure would have been applied: statistical analysis paths would be compared and the reason for the differences would be sought. In case of an error in one of the analyses the more accurate analysis would have been reported.

In the fifth stage all authors (A, B, C, D) were involved in the process of report writing. It was intended that in the final report the identical findings provided by both statisticians (C and D) would be presented only once. In contrast the findings, which disagreed or complemented each other would be described separately. No additional statistical analyses were permitted after the fourth stage. However, an appropriate reformatting (adjustment) of graphs and tables was allowed. The data and the source code used can be obtained under request from the authors.

In the original final study report both statistical contributors wrote their own statistical methods sections, two results sections and two separate discussions concluding by a conjoint discussion. For the sake of compactness only a concise summary of the original final report is provided below.

### 2.3. Statistical Methods

Data were visualized in two different ways. Statistician C preferred boxplots whereas statistician D favored histograms with superimposed density functions. As far as the distributional assumption is concerned statistician D considered only the Weibull distribution. In contrast, statistician C computed adjusted Anderson–Darling (AD) goodness-of-fit estimates and probability plots in order to clarify the true sampling distribution [11]. Finally, statistician C concentrated on the two-parameter Weibull and the Normal distributions.

Estimation techniques applied by both statisticians differed as well. Statistician C fitted the parameters of the Weibull and Normal distributions by both Least Squares (LS) and Maximum Likelihood (ML) techniques [6,13]. For LS fit the median rank (Benard) default assumption in Minitab was used [6]. The corresponding 95% confidence intervals (95% CI) were computed. In contrast, statistician D provided Weibull parameters LS estimates according to the suggestions in [13] for *n* = 40. Statistician D applied two competing methods: regression of XonY together with median ranks (XonY/median) and regression of YonX with hazen ranks (YonX/hazen). 95% CI were calculated for all estimates according to the procedure suggested in [13] (termed Menon 95% CI).

Statistician C tested Weibull parameters for differences with permutation tests programmed in *R* [27]. A permutation test generates a reference distribution under the null hypothesis (H_0_, no difference between groups) by randomly rearranging group labels and computing the value of the test statistic for a lot of such rearrangements. The reference distribution represents values that are plausible under H_0_. The value of the test statistic actually observed is compared to the reference distribution and *p*-values are calculated as the fraction of cases in the reference distribution that show a value at least as extreme as the one actually observed. In this study *R* = 10,000 permutations were performed and the test statistic was the difference of estimated Weibull parameters between different groups (m_i_ − m_j_ and s_i_ − s_j_). It is zero under H_0_ (parameters are the same) and larger differences provide more evidence against H_0_. The test for m was always done first since the test for s requires homogeneity in m and was only performed if no significant difference in m was found. In order to compare Weibull parameters among more than two groups a global test was developed. The mean absolute differences between Weibull parameters estimated in all groups under comparison were used as test statistic (mean|m_i_ − m_j_| and mean|s_i_ − s_j_|). If significant differences were found in the global test, pairwise comparisons between all groups were performed, including correction for multiple comparisons. Under H_0_ the probability of at least one significant result in multiple comparisons (family-wise error rate, FWER) will be larger than the nominal type I error rate α (typically α = 5%). A simple method to control FWER is the Bonferroni-Holm [28] method in which the smallest *p*-value is multiplied by the number of individual tests (k), the second smallest by k-1 and so on (with the restrictions that the initial order of *p*-values is kept and all *p*-values < 1), and evaluated at the initial level α:

ordered *p*-values: p_1_ ≤ p_2_ ≤ ... ≤ p_k_
adjusted *p*-values: p_i_^adj^ = max_j≤i_ [(k-j + 1) p_j_]_1_
where [x]_1_ = min(x,1) and i = 1, 2, ..., k

Such Bonferroni-Holm adjusted *p*-values are reported when multiple comparisons are performed.

In contrast, statistician C conducted equal shape (standard deviation) and equal scale (mean) Bartlett’s modified likelihood ratio tests together with the appropriate Bonferroni post-hoc confidence interval [6,8] with Minitab Version 14 [29]. The impact of SP and ZM factors was evaluated by a general test looking for differences in parameters between all six tested G groups. Results of statistical analyses with *p* < 0.05 were interpreted as statistically significant.

## 3. Results

**Figure 2:**
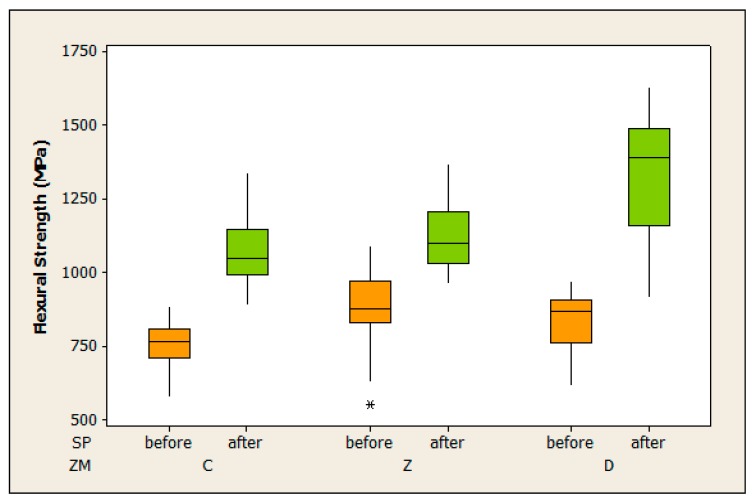
Boxplots for the biaxial flexural strength in each tested group G and ZM/SP levels.

**Figure 3:**
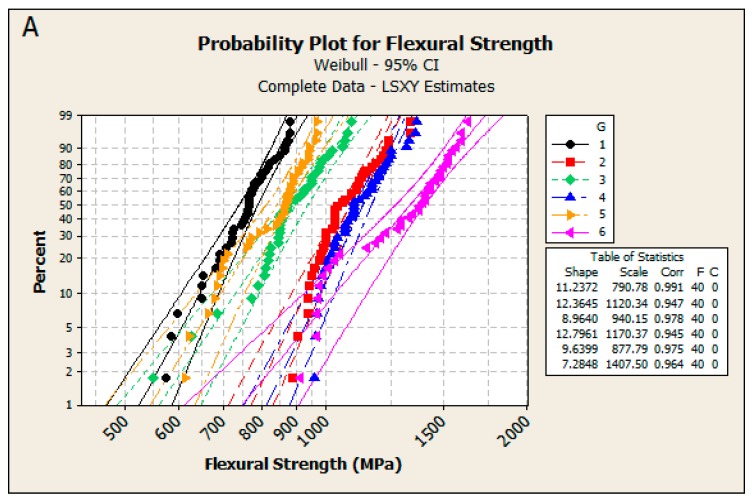

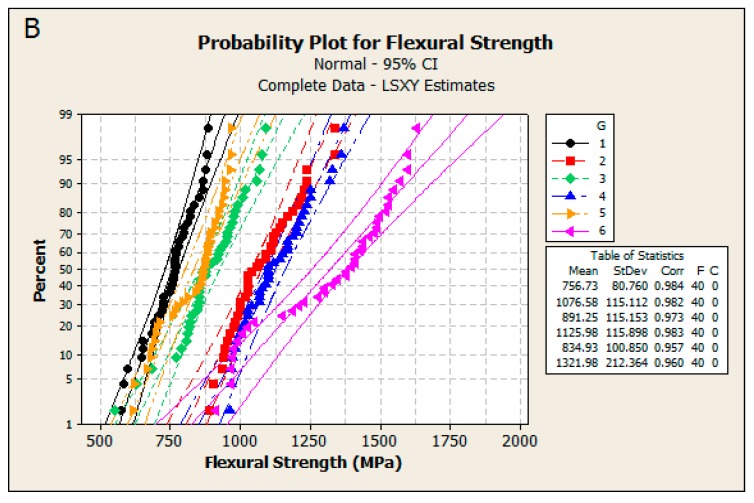
Probability plots for biaxial flexural strength in each tested group G for Weibull LS (**A**) and Normal LS (**B**).

Figure 2 shows the distributions of the biaxial flexural strength observations in each tested group. Probability plots for Weibull and Normal assumptions are depicted in Figure 3.

The Anderson-Darling goodness-of-fit estimates for the two-parameter Weibull and Normal distributions showed that the Weibull assumption was better than the Normal one for all groups with exception of C/after and Z/after (see [3] Table 4). Although Anderson-Darling estimates for Weibull and Normal distributions differed between LS and ML fitting methodologies, their suggestions for the better fit were consistent. While probability plots in Figure 3 indicated discrepancies from straight lines it was impossible to find any alternative uniformly optimal fitting sampling distribution in all tested groups.

Generally, biaxial flexural strength observations appeared to be larger in groups G2, G4 and G6 within SP = “after” condition. Variance of strength values was small in G1, at a medium level in G2 to G5, and large in G6 (Table 2, Figure 2).

The descriptive statistics for the Weibull distribution are shown in Table 3. As the Weibull parameters estimates obtained by the XonY/median and Benard LS approaches were comparable to the YonX/hazen one, only the later results are reported. For completeness the ML estimates are provided.

Results obtained by Weibull and Normal estimation methods were comparable. Generally, larger mean biaxial flexural strength values resulted in larger estimates for s and lower variance of the biaxial flexural strength in larger estimates for m. Estimates for s were e.g., higher in the groups with rather large outcomes (G2, G4, G6). The large variance in G6 resulted in small m, the low variance in G1 in a rather large m. Generally, G2 and G4 appeared to be very similar, characterized by large s and m. G3 and G5, in contrast, were characterized by both small s and m. Taking 95% CI into account, differences in s compared to m were more prominent. In particular, s appeared to be much higher in groups G2, G4 and G6 (with after SP).

**Figure 4:**
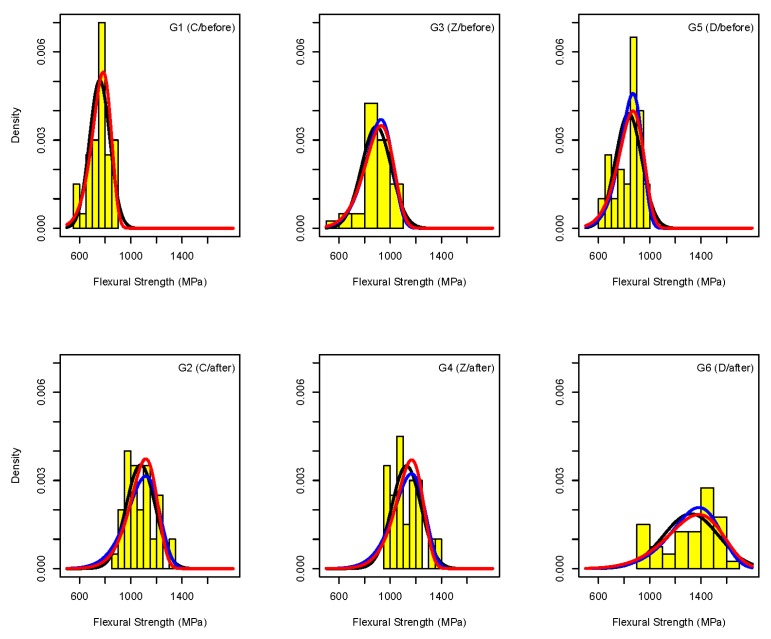
Histogram of biaxial flexural strength with superimposed densities: Weibull estimated by ML (**blue**) and YonX/hazen (**red**) and Normal (**black**). Factor G and levels of ZM/SP are indicated on top of each plot.

There were differences in Weibull estimates obtained by the ML and LS (YonX/hazen) approaches (Table 3). Their relevance is visualized in histograms with superimposed Weibull and Normal density functions in Figure 4.

Permutation tests were applied to test for H_0_ that there are no differences between Weibull parameters in the groups of interest. Tests for differences in s were only made if no differences in m were found, since they rely on homogeneity in m. As the results of the permutation tests applied to XonY/median and YonX/hazen estimates were comparable, only the latter are reported in Table 4.

With respect to the impact of SP significant differences were found for zirconia Z between G3 and G4 (*p* = 0.037) as well as for zirconia D between G5 and G6 (*p* < 0.0001) (Table 4a). As there was no evidence against homogeneity in m for zirconia C, s was analyzed and found to differ significantly (*p* < 0.0001).

In order to analyze the effect of ZM comparisons of groups with constant SP were made (Table 4b). Significant differences in m were found between groups G2-G4-G6 (C-Z-D/after) (*p* < 0.0001). Accordingly, s was only tested for G1-G3-G5 (C-Z-D/before) and found to differ significantly (*p* < 0.0001).

As the global test for m was significant for G2-G4-G6 (C-Z-D/after), all pairwise comparisons G2-G4, G2-G6, G4-G6 were computed (Table 4c). Significant differences after Bonferroni-Holm correction for multiple testing were found for G2-G6 and G4-G6. The non-significant G2-G4 comparison for m (*p* = 0.821) was tested for differences in s and did also not reach significance (*p* = 0.084). Pairwise comparisons for s were also made in the groups G1-G3-G5 (C-Z-D/before), as the global test for s was significant. All of the possible pairwise tests were significant after Bonferroni-Holm correction (Table 4c).

Alternatively, the general analysis comparing all six tested G groups found that for Weibull (LS) modulus m6 < (m2, m4) but (m1, m3, m5, m6) and (m1, m3, m5, m2, m4) (*p* = 0.006) and characteristic strength s1 < (s3, s5) < (s2, s4) < s6 (*p* < 0.001). In contrast, for Weibull (ML) modulus no differences between moduli in all six factor levels were found (m1, m3, m5, m2, m4, m6) (*p* = 0.409) and the findings for the characteristic strength agreed with those for LS fitting technique leading to s1 < (s3, s5) < (s2, s4) < s6 with *p* < 0.001.

The results of the tests for LS and ML fitting techniques conducted under Normal sampling distribution assumption agreed perfectly well leading to sd6 > (sd1, sd3, sd5, sd2, sd4) (*p* < 0.001) and mean1 < (mean3, mean5) < (mean2, mean4) < mean6 (*p* < 0.001).

## 4. Discussion

Strictly speaking the Weibull distributional assumption is only a special case of a more general approach to strength distributions [5]. Weibull assumption is preferred in practice due to its flexibility. It provides nice fits to strength data [5,6]. It has also strong probabilistic foundations for strength measurements. Although the Weibull distribution is more appropriate to analyze strength data of brittle materials than the Normal one [5], the Normal one is frequently used due to its convenience and availability in general-purpose statistical programs. It is difficult to discern the appropriateness and implications of Weibull and Normal assumptions in practice, notably, for small sample sizes [5].

As a rule of thumb at least 30 measurements in each tested group are necessary to be able to recognize the true sampling distribution at all [11]. Estimation of the scale (s) of the two-parameter Weibull distribution and mean of the Normal one requires fewer observations than the estimation of modulus (m) and sd, respectively [9]. Although Nohut [7] shows that for sample sizes below 150 per group no clear discrimination between Weibull and Normal sampling distributions is possible.

Abernethy [11] suggested a conservative approach to Weibull analysis. He recommended application of a two-parameter Weibull distribution as a working assumption irrespective of curvature in the probability plots. Given the respectable number of 40 observations in each group statistician C investigated the true underlying sampling distribution and found that no uniformly optimal fitting distribution could be suggested. The two-parameter Weibull distributional assumption was in 4 out of 6 tested groups better than the Normal one for the data at hand. Therefore, we considered both Weibull and Normal distributional sampling distributions for data analysis. We think that the dilemma of discerning Weibull and Normal distributions will stay an unsolved problem. As neither Weibull nor Normal distributional assumptions fit the data in all tested groups perfectly well, the truth seems to lie somewhere in between. In this respect our approach is an extension of the Abernethy’s [11] conservative approach to two different distributional assumptions. The use of two different distributional assumptions protects us from being over-optimistic.

Our analysis indicated a strong evidence for the relevance of both ZM and SP on the biaxial flexural strength values. Generally, SP compared to ZM had more impact on mean biaxial flexural strength and estimated scale s than ZM. Wet polishing after sintering appears to generally increase s. Within this group, C and Z zirconia materials were not distinguishable and D reduced modulus m. However, a clear pattern for m was not observed and it seems to be influenced by the combination of ZM and SP. In particular, the combination in the group G6 (D/after) results in small m and a high variance in biaxial flexural strength. Assuming large values for both s and m would be beneficial, the combination in groups G2 (C/after) and G4 (Z/after) performed best.

After unblinding the project, we realized that the treatment of the specimens preparation in clusters might actually led to problems for group G6 (D/after). We were unable to identify the real cause. However, probability plots (Figure 3) and histograms (Figure 4) clearly indicate that the distribution in G6 (D/after) is bi- rather than uni-modal suggesting that possibly a cluster of specimens in this group had different properties. Interestingly, this cluster led to higher sd (lower m) estimates in G6 (D/after) provoking our recommendation not to use this technique in practice.

Moreover, the following additional potential uncertainties for Table 1a have been identified in the main experiment [3]: material (manufacturer, lot number), sintering temperature inaccuracy (unequal temperature inside the oven, too early opening of the oven’s door), measurement error (micrometer screw precision), positioning within the testing device (splinters), measurement error of the testing machine for biaxial flexural strength measurements. Due to the oven or the polishing machine capacity specimens were frequently handled in clusters. If an inaccuracy occurred then all of the specimens in a cluster were possibly affected by it.

We obtained discordant decisions from the global Weibull test depending on the estimation method. LS indicated that there is a difference in moduli between the six tested groups. In contrast, ML found no evidence for any differences. One possible approach to dissolve this discrepancy would be to look closer at the relevance of the differences. According to Nelson [9] the sample size of 40 observations in each tested group implies that if the true modulus equals 10, then with probability 99.5% the modulus estimates should be found in interval (6.2, 16.0). Interestingly, modulus estimates in Table 3 can actually be found in this interval. This argument suggests that there are no genuine differences in moduli between the six tested G groups supporting the Weibull/ML finding. A second argument trying to dissolve the LS/ML discrepancy would be to apply a more stringent significance level 0.005 instead of the inappropriately high but common 0.05 one as suggested by Johnson [30]. Johnson’s suggestion aims for increasing reproducibility of the reported findings in the scientific literature. Application of a lower α = 0.005 level for testing would imply an agreement of the global conclusions from LS/ML estimation techniques for the Weibull distributional assumption: there would be no evidence for differences in moduli between the six groups and the characteristic strengths would be ordered G1 < (G3, G5) < (G2, G4) < G6. For the Normal assumption the ordering of the means would agree with that for Weibull characteristic strengths. However, the standard deviation in G6 would be larger than standard deviations in all other tested groups. In this respect Weibull and Normal findings would still disagree.

Perhaps it is a misperception that the results of a statistical analysis have to be unique. We suggested two independent blinded statistical analyses by two statisticians who were unaware of the factor level’s assignment and the meaning of the primary outcome. Our primary goal was not to obtain identical results but rather to get a better picture of the underlying truth. Therefore, we deliberately decided not to apply any rigid pre-specified guidelines for statistical analysis, any detailed analysis plan including rigorously predefined objectives and any inflexible specifications of the statistical methods for the primary endpoint prior to the study.

Gøtzsche [17] apprehends data analysis as a highly subjective process vulnerable to bias and suggests that actually two manuscripts should be written and both manuscripts must be completed and approved by the authors before the code is broken. We rather aimed for a single final report containing the identical results with additional sections describing explicitly possible discrepancies. We found that it is easier for a reader to concentrate on the main findings and grasp the differences.

It was the first time we applied “independent statistical analyses” and “parallel manuscript writing” to a “blinded data set”. The “blinding of the data set” was very unfamiliar to both study supervisor A, as well as to the statisticians C, D. Usually, A would supply a statistician with unlimited information about the measurements and the experiment properties prior to the statistical analysis. On the other hand, both statisticians would ask for as much background information as possible about the meaning of the variables and the expected effects prior to the data analysis in order to correctly understand the data and to provide optimal statistical analysis for the project at hand. “Blinding of the data set” technique was clearly in conflict with our daily statistical consultation experience. Our experiment forced us, however, into an unusual situation and possibly inspired a more impartial statistical analysis.

The “independent statistical analyses” had definitely a considerable influence on both statisticians. They provided a more watchful analysis, tended to spontaneous self-verification and an increased alertness by the simple fact that the results would be verified and critically discussed by an independent statistician. We experienced that “independent statistical analysis” is a very powerful tool to ensure good statistical analysis. It is even stronger than a simple correctness check of the reasoning path provided by a statistician. It was a very inspiring experience. It led to a deeper and more thorough analysis. To our surprise both independently working statisticians went through two differing analysis paths for data visualization, estimation and testing and arrived at complementary results.

The “parallel manuscript writing” technique was also new for us. It forced both statisticians to be clear about their final conclusions, to tie themselves down to one explicit ultimate interpretation and to present concisely their findings in tables and figures. Both independently written manuscripts could be conveniently merged into one final report at the fifth stage of our study (Figure 1).

Given two parallel drafts the final writing of the report concentrated on collation of both abstract, results and discussion sections, notation unification and consolidation of tables and figures. We had to shorten the exposition and to counteract repetitions. For a similar future study, it would be beneficial to agree on the notation beforehand. Furthermore, it would be advantageous to put a limit on the length of paragraphs written independently by biostatisticians.

One possible limitation of a blinded statistical analysis is its inefficiency [17]. There is extra work needed to conduct statistical analysis twice and to produce two independent drafts. Miller & Stewart [23] point out that a requirement for the biostatistician to remain blinded adds a level of complexity to the study implementation. When planning the amount of time for data analysis we followed suggestions by Pocock [18,31] who stressed that one should allow enough time for analyzing the measurements as good-quality statistical analysis cannot be achieved overnight so that an adequate provision of time for the analysis and interpretation of trial data should be recognized when planning a study. Therefore, we warranted both data analysts 3 weeks for the data analysis and their independent writing of “Results”, “Summary”, “Conclusions” and “Rationale for the choice of statistical methods” sections. In fact, each of the statisticians spent at least 25 h for analysis and draft writing. We feel that the time interval of 3 weeks provided an appropriate amount of time for a deepened statistical analysis. We admit that the expenditure of time was much increased as compared with a common non-parallel statistical analysis. In our case, however, such an effort was justified.

The statistical analysis paths took by the statisticians differed. Statistician D preferred the permutation tests approach for Weibull statistics. This innovative idea is motivated by the fact that permutation tests are non-parametric, allow for any arbitrary test statistic and do not make any assumptions about the distribution of this test statistic under the null hypothesis (H_0_, no difference between groups). It can be programmed in R and is independent from sophisticated statistical software [6,11,12]. One deficiency of the approach was that the permutation tests for post-hoc tests in Table 4c had to be programmed separately and the application of the Bonferroni-Holm correction for the pairwise tests (Table 4c) was necessary. Interestingly, statistician D arrived at different conclusions mainly by not applying any global analysis. Separate comparisons in each factor level suffered from several drawbacks such as multiple comparisons, difficult interpretation and complicated design. An ANOVA framework including both factors would be better suited for the specific problem. However, normally distributed variables with equal variances would be required. Methods form transformation of Weibull distributed into normally distributed variables are available and could be a promising alternative [32].

The analysis path suggested by statistician C considered both Weibull and Normal distributions using the graphical and statistical facilities available in Minitab. It applied global likelihood ratio tests together with integrated post-hoc confidence intervals. No additional programming was required. Finally, this approach was considered for the analysis of the larger data set with a greater number of tested groups in [3].

Table 5 contains the final workflow for a pragmatic statistical analysis of flexural strength data developed in our study and successfully applied in [3]. It consists of seven steps starting with visualization of the measurements (Step 1), choice of at least one reasonable distributional assumption (Step 2), estimation of the parameters of a distribution (Step 3), check of the goodness-of-fit (Step 4) and estimation of 95%CI (Step 5). In order to understand which tested groups are better than others in terms of location and spread we have to apply appropriate statistical tests (Step 6). These tests require a specification of a single distribution applicable to all tested groups. In practice, however, it is quite impossible to find a single distributional assumption fitting all groups equally well. Therefore, we extended Abernethy’s [11] conservative approach to two different reasonable distributional assumptions (Weibull and Normal in our case) as a working hypothesis (Step 2). As a consequence, we have to compare the results obtained under differing distributional assumptions (Step 7).

We did not intend to cover the whole range of possible statistical approaches for the analysis of the strength data. We admit that the true range of the varying inter-personal statistical approaches is not well reflected by just two participating statisticians. For example, some researchers might opt for an equivalent volume approach within the Weibull analysis [5,10]. Others might consider a log-normal distributional assumption for the data analysis instead of Weibull or Normal ones. They would argue that the log-normal distribution may be a useful alternative for stabilizing and reducing variance as well as giving a sensible linearization of probability plots. An ordinary ANOVA applied to lognormal data may be robust enough to answer the questions in a very rapid and straightforward manner.

Despite these possible shortcomings, our blinded approach was a very helpful tool at the stage of finding a practicable statistical method for the final data analysis in [3]. It complements the recommendations by Hannigan and Lynch [33] and should definitely be taken into consideration.

## 5. Conclusions

Within the limitations of this investigation it can be concluded that:

- Zirconia specimen preparation method has an impact on characteristic strength (s) and mean of the biaxial flexural strength but in majority of tested groups practically no relevant impact on modulus (m) and standard deviation (sd) of the results.
- All three tested zirconia materials showed different characteristics strengths and mean flexural strength results. Group G6 (D/after) showed higher spread leading to smaller modulus and increased sd estimates.
- The “blinded data set”, “independent statistical analyses” and “parallel manuscript writing” techniques had an influence on the findings for strength data. The impact of “independent statistical analyses” was most pronounced.
- Statistical analysis paths taken by both independently working biostatisticians differed.
- The major difference in the findings was caused by two alternative distributional assumptions (Weibull/Normal) and alternative estimation methods (LS/ML).

## Figures and Tables

**Table 1:** Sources of Uncertainty.

Study Phase	Uncertainty Source
Data generation (a)	Specimens/Subjects Investigators Data collectors and managers Precision of measuring devices Outcome assessors
Statistical data analysis (b)	Sample size Data analyst Descriptive statistics Assumption on the sampling distribution (model uncertainty) Outliers (data uncertainty) Choice of the statistical approach (frequentist or Bayesian) If Bayesian, then prior elicitation (prior uncertainty) Transformation of variables Parametric or non-parametric analysis Tests/Confidence intervals Choice of the estimation technique within the approach chosen Interpretation of the results Missing data handling Subgroup analysis Covariates selection
Writing of the manuscript (c)	Choice of the findings to report on Choice of the graphs to be shown Manuscript writer

**Table 2:** Data summary for Normal assumption. n: number of observations; q1: first quartile; q2: third quartile.

Tested Groups	ZM	SP	n	Min	q1	Mean	Median	q2	Max	sd
G1	C	before	40	575	719	757	765	804	884	79
G2	C	after	40	890	997	1077	1050	1143	1340	113
G3	Z	before	40	551	842	891	878	966	1090	115
G4	Z	after	40	962	1030	1126	1100	1203	1370	114
G5	D	before	40	615	764	835	869	908	969	102
G6	D	after	40	915	1180	1322	1390	1490	1630	214

**Table 3:** Point and interval estimates of the Weibull parameters modulus (**m**) and scale (**s**) based on ML or YonX/hazen, respectively.

Tested Groups	ZM	SP	Method	m	95% CI (m)	s	95% CI (s)
G1	C	before	ML YonX/hazen	11.4 11.4	[8.9, 14.6] [8.2, 15.9]	791 791	[768, 814] [768, 814]
G2	C	after	ML YonX/hazen	9.6 11.4	[7.6, 12.0] [8.2, 15.9]	1129 1126	[1090, 1168] [1093, 1159]
G3	Z	before	ML YonX/hazen	9.4 8.9	[7.3, 11.9] [6.4, 12.4]	939 942	[906, 972] [907, 977]
G4	Z	after	ML YonX/hazen	10.3 11.8	[8.1, 13.0] [8.5, 16.3]	1178 1176	[1141, 1217] [1143, 1210]
G5	D	before	ML YonX/hazen	10.9 9.5	[8.3, 14.1] [6.8, 13.2]	877 880	[851, 904] [849, 911]
G6	D	after	ML YonX/hazen	7.9 7.0	[6.1, 10.3] [5.0, 9.7]	1409 1414	[1352, 1468] [1348, 1484]

**Table 4:** Permutation tests for differences in Weibull parameters estimated by YonX/hazen. (**a**) Differences between two levels of SP within each level of ZM. Test statistic: **m_i_ − m_j_** and **s_i_ − s_j_**; (**b**) Global test for differences between the three levels of ZM within each level of SP. Test statistic: mean|**m_i_ − m_j_**| and mean|**s_i_ − s_j_**|; (**c**) Pairwise tests to B for differences between three levels of ZM within each level of SP. Test statistic: **m_i_ − m_j_** and **s_i_ − s_j_**. *p*-values were adjusted by the Bonferroni-Holm method.

	Comparison	Condition	Test Statistic		*p*-Values	
			m	s	m	s
(a)	before-after (G1-G2)	C	0.014	−334.9	0.9830	<0.0001
	before-after (G3-G4)	Z	−2.831	−234.4	0.0370	NA
	before-after (G5-G6)	D	2.494	−534.7	<0.0001	NA
(b)	C-Z-D (G1-G3-G5)	before	1.664	100.4	0.2010	<0.0001
	C-Z-D (G2-G4-G6)	after	3.186	192.4	<0.0001	NA
(c)	C-Z (G1-G3)	before	2.496	−150.6	NA	<0.0001
	C-D (G1-G5)	before	1.948	−88.8	NA	<0.0001
	Z-D (G3-G5)	before	−0.547	61.8	NA	0.0080
	C-Z (G2-G4)	after	−0.350	−50.2	0.8210	0.0840
	C-D (G2-G6)	after	4.428	−288.7	<0.0001	NA
	Z-D (G4-G6)	after	4.778	−238.5	<0.0001	NA

**Table 5:** A recommended statistical analysis workflow for flexural strength measurements. If not otherwise indicated [29] can be used (path: Stat/Reliability-Survival/Distribution Analysis (Right Censoring)/Parametric Distribution Analysis/).

Step	Decision/Action
Step 1	**Data visualization in each tested group** (see Figure 2 and Figure 4): Check data visually by histograms, scatterplots and/or boxplots. Are there any outlying observations? If yes, check if they are possibly typing errors and correct them. Are histograms approximately symmetric in each tested group? If no, you may try to transform the measurements.
Step 2	**Distributional assumption for measurements** (see Section 1.1): Do you think that each measurement consists of a possibly large number of independent random fluctuations? If yes, go for a Normality assumption directly (for approximately symmetrical histograms) or after a (logarithmic) transformation of measurements. Do you believe in the “weakest link” process generating your data? If yes, go for a Weibull assumption. If both assumptions seem to be reasonable use both Normal and Weibull distributional assumptions for your working hypothesis.
Step 3	**Descriptive statistics: Estimation of parameters in each tested group:** Under Normality assumption: compute mean and standard deviation (sd). Under Weibull assumption: compute the characteristic strength (s) and modulus (m). See the open source Excel-calculator (Appendix C in [13]). Remember: (s “=” mean) and (m “=” 1/sd) (see Section 1.1)
Step 4	**Check the goodness-of-fit in each tested group:** Compute the goodness-of-fit estimates. Generate probability plots (see Figure 3) and check if they are linear. In case of approximate linearity the assumed distribution fits the data well. In case of clear non-linearity interpret the results with caution.
Step 5	**Estimation of 95%CI for parameters in each tested group:** Under Normality assumption: 95% CI (mean) and 95% CI (sd). Under Weibull assumption: 95% CI (s) and 95% CI (m). See the open source Excel-calculator (Appendix C in [13]).
Step 6	**Are there any differences between tested groups?** Normal mean: Apply an Analysis of Variance (ANOVA). Normal sd: Apply a Levene-Test. Weibull s and m: Apply the Bartlett’s modified likelihood ratio tests.
Step 7	**Check the results:** Critically check if the results obtained in Step 6 agree with the graphs generated in Step 1. If you applied both the Normal and the Weibull assumptions critically check if the results obtained in Step 6 are comparable. Remember: (s “=” mean) and (m “=” 1/sd) (See Section 1.1).
